# Mitochondrial Involvement in the Adaptive Response to Chronic Exposure to Environmental Pollutants and High-Fat Feeding in a Rat Liver and Testis

**DOI:** 10.3390/cells8080834

**Published:** 2019-08-05

**Authors:** Vincenzo Migliaccio, Ilaria Di Gregorio, Rosalba Putti, Lillà Lionetti

**Affiliations:** 1 Department of Chemistry and Biology “Adolfo Zambelli”, University of Salerno, 84084 Fisciano, Italy; 2 Department of Biology, University of Naples, Federico II, 80126 Naples, Italy

**Keywords:** DDE, high-fat diet, mitochondrial UCP2, ROS, antioxidant system

## Abstract

In our modern society, exposure to stressful environmental stimuli, such as pollutants and/or chronic high-fat feeding, continuously induce tissular/organ metabolic adaptation to promote cellular survival. In extreme conditions, cellular death and tissular/organ damage occur. Mitochondria, as a cellular energy source, seem to play an important role in facing cellular stress induced by these environmental stimuli. On the other hand, mitochondrial dysfunction and oxidative stress play a key role in environmental stress-induced metabolic diseases. However, little is known about the combined effect of simultaneous exposure to chronic high-fat feeding and environmental pollutants on metabolic alterations at a tissular and cellular level, including mitochondrial dysfunction and oxidative stress induction. Our research group recently addressed this topic by analysing the effect of chronic exposure to a non-toxic dose of the environmental pollutant dichlorodiphenyldichloroethylene (DDE) associated with high-fat feeding in male Wistar rats. In this review, we mainly summarize our recent findings on mitochondrial adaptive response and oxidative stress induction in the liver, the main tissue involved in fat metabolism and pollutant detoxification, and in male gonads, the main targets of endocrine disruption induced by both high-fat feeding and environmental pollutants.

## 1. Introduction

Mitochondria are the main organelles involved in cellular energy production and play a key role in facing cellular needs in response to external stimuli. Environmental stimuli, such as pollutants and/or chronic high-fat overnutrition, are very frequent in modern society and may induce metabolic diseases by acting on the mitochondrial function and oxidative stress induction.

Several recent scientific studies have highlighted that environmental pollutants generate cellular toxicity in different animal species, including humans. It is well-known that hydrophobic chemicals, such as polychlorinated biphenyl compounds (PCBs), persist in the environment for a long time and are also detectable across the world at a long distance from the utilization site [1,2,3]. Dichlorodiphenyltrichloroethane (DDT) represents one of the well-known and most used organochlorines (OC) belonging to the PCBs family. This chemical was synthesized for the first time in 1874 by Othmar Zeidle, but it became famous in 1939, when the chemist Paul Hermann Müller discovered DDT’s proprieties as poisonous against arthropod vectors of parasites [4]. In the last century, DDT was used during the second World War and post-World War period to control the extension of malaria cases. The massive and uncontrolled use of DDT briefly produced negative effects on the environment, such as the thinning of eggshells in birds [5,6] and reduction of hatching [7]. Moreover, negative implications were also observed in reproduction in higher animals [8]. Therefore, DDT use was banned in many countries and restricted to equatorial zones where malaria is still endemic [9]. However, DDT and its metabolites, as other persistent organic pollutants (POPs), can migrate through the atmosphere as gases and aerosols moving thousands of kilometers from the point of release [10]. Moreover, given their liposolubility, these substances undergo bioaccumulation and biomagnification phenomena, with an increase in concentration for the species at the top of the food chain [11]. Several recent studies underlined that DDT and its metabolites are able to induce metabolic alterations and disfunction in different organs and tissues. For example, it has been shown that DDT and its major metabolite dichlorodiphenyldichloroethylene (DDE) promote diabetes, hepatic morphological alterations in terms of toxicant-associated steatohepatitis (TASH), and endocrine disorders that compromise reproductive efficiency [12,13,14]. In addition, DDT and DDE were found to be directly implicated in reactive oxygen species (ROS) over-production, cellular oxidative stress, and apoptosis onset in vivo and in vitro by modulating the mitochondrial function [15,16]. It is noteworthy that fat-soluble chemicals are easily introduced in organisms through contaminated food and their intake may increase during high-fat feeding. Fats represent a further point of interest for scientific research. It is well-known that high lipid consumption induces metabolic alterations, leading to the development of obesity and its related metabolic diseases, such as cardiovascular diseases, hepatic steatosis, and insulin resistance/diabetes [17,18,19,20,21,22,23]. It is noteworthy that a high-fat diet (HFD) elicits several metabolic disorders, including mitochondrial dysfunction and oxidative damage [24,25,26,27,28,29,30]. Mitochondrial function and its adaptation to dietary components represent a key point in metabolic research. In fact, HFD has been suggested to increase ROS production and induce mitochondrial dysfunction, generating metabolic alterations that, in turn, could lead to the development of HFD-induced insulin resistance [31,32]. However, little is known about the combined effect of simultaneous chronic high-fat feeding and environmental pollutant exposure on metabolic alterations at a tissular and cellular level, including mitochondrial dysfunction. This aspect could clarify whether dietary fat could act as a vehicle for the pesticide, suggesting a possible synergistic effect of the two different stimuli. Our research group recently addressed this topic by analysing the effect of chronic exposure to a non-toxic dose of the environmental pollutant DDE combined with a low-fat or high-fat diet in male Wistar rats, focusing in particular on the liver and testis function, as well as on the mitochondrial adaptive response [33,34]. In this mini review, we summarize our findings on metabolic responses in the liver, which represents the main tissue involved in energetic metabolism and detoxification, as well as in male gonads, which represent one of the principal targets of endocrine disrupter chemicals [35]. It is interesting to analyse how different organs respond to both overfeeding and environmental pollutant exposure, in order to observe whether common cellular mechanisms are involved. Our experimental design utilized adult male Wistar rats subdivided into four different groups: control animals, fed a standard laboratory diet (N group); DDE-treated animals, fed a standard diet associated with DDE administration (N + DDE group); high-fat-fed animals that received HFD (45% fat, D group); high-fat + DDE-treated animals that received simultaneous exposure to HFD and DDE (D + DDE group). Pesticide (10 mg/Kg b.w.) was administrated every day, orally, during a period of 28 days. In liver samples we analysed lipid accumulation, antioxidant system activity, mitochondrial function and cellular detoxification markers [34]. At a testicle level, we mainly focused our attention on seminiferous tubules, spermatogenesis compromising, oxidative stress, apoptosis and cellular proliferation [33].

In the first part of the present review, we introduce the role of mitochondria and oxidative stress in cellular homoeostasis. Then, we focus on the effect of HFD and DDE to a single exposure or a simultaneous exposure on hepatic metabolism and mitochondrial involvement. In the last part of the review, we focus on testis adaptation to HFD and DDE, as single exposure or simultaneous exposure. No-synergic effect of HFD and DDE was found in either the liver or testis.

## 2. Mitochondria and Oxidative Stress Generation in Biological Systems

Mitochondria are important organelles involved in different metabolic processes in cells. They play a key role in energy production, regulating several cellular physiological pathways, such as differentiation, proliferation, and programmed death [36]. These mechanisms are regulated by both mitochondrial and nuclear gene expression [37]. It is known that several mitochondrial proteins and subunits are encoded by nuclear genes, known as nuclear-encoded mitochondrial genes (NEMG). Moreover, accumulative evidence suggests that mitochondrial factors act as signals to regulate the expression of nuclear genes. The factors produced by nuclear activity regulate mitochondrial function, creating a crosstalk mechanism between mitochondria and the nucleus [37]. In this context, mitochondria–nucleus crosstalk represents an important physiological process that, if altered, permits mitochondrial dysfunction and/or cellular damage to take over. These two communication pathways are able to promptly satisfy cellular requests in response to metabolism alterations and exposure to environmental factors [38]. Moreover, mitochondrial-generated ROS and metabolites act as signaling molecules and cofactors that regulate fundamental nuclear processes. Excessive ROS production can induce cellular oxidative stress and damage cellular components and macromolecules, including DNA [39].

Oxidative stress represents a condition observed in biological systems due to the imbalance between ROS production and endogenous antioxidant systems useful to counteract oxidative damage. ROS are produced in different cellular districts and include radical and non-radical chemical species: (a) superoxide anion (^•^O_2_^−^), which is mainly produced at a mitochondrial level; (b) peroxyl radicals (R-OO^•^), which derive from the oxidative damage in membrane lipids; and (c) hydrogen peroxide (H_2_O_2_), which is produced as a consequence of superoxide-catalyzed dismutation by superoxide dismutase activity (SODs). Several studies have shown that the main source of ROS production is represented by mitochondria [40,41,42], where different sites of superoxide/hydrogen peroxide generation have been identified [43]. Most of these sites deliver their superoxide/hydrogen peroxide product exclusively to the mitochondrial matrix, but at least two of the sites (Complex III of the electron transport chain and mitochondrial glycerol 3-phosphate dehydrogenase) deliver superoxide to both the matrix and the cytosolic face of the inner membrane, because they are situated on the outer side of the mitochondrial inner membrane [44,45,46,47]. These different site-specific topologies in the delivery of superoxide/hydrogen peroxide to different compartments have been suggested to be of great importance in redox signaling. Indeed, ROS have also been shown to act as signaling molecules [48]. It has been hypothesized that redox signaling by the superoxide generated by complex III in the cytosol will be very different from redox signaling by the superoxide generated in the mitochondrial matrix by other sites [47].It is interesting to underline that recent works have shown that increasing levels of superoxide, through treatment with the pesticide paraquat, result in an increased lifespan, and that ROS have a compartment-specific effect on the lifespan [49,50]: elevated ROS in the mitochondria act to increase the lifespan, whereas elevated ROS in the cytoplasm decrease the lifespan [51]. A further ROS source is represented by cytochrome P450 (CYP450) activity in mammalian species [52]. CYP enzymes catalyze the oxygenation of an organic substrate with the simultaneous reduction of molecular oxygen. This mechanism is involved in the xenobiotics-induced cellular detoxification process. ROS can be released by the P450 reaction cycle under conditions in which the transfer of oxygen to a substrate is not tightly controlled [52]. High ROS production induces oxidative damage in lipids and other macromolecules, generating metabolic alterations and cellular diseases. In physiological conditions, ROS levels can be regulated by antioxidant enzymes or antioxidant molecules which are able to quench radical species to limit oxidant process propagation. In this way, ROS levels remain low in cells, playing a key role in regulating cellular metabolism, proliferation, and death [53,54]. On the other hand, increased ROS levels are associated with metabolic dysfunction [55,56] and inflammation. Noteworthy, metabolic dysfunction and inflammation have also been suggested to be associated with xenobiotics exposure [57,58,59]. At a mitochondrial level, the superoxide may be reduced using antioxidant activity. In fact, superoxide released in the matrix or in the mitochondrial inner membrane space is converted into H_2_O_2_ by superoxide dismutases (SODs). In the mitochondrial matrix, GPx reduces H_2_O_2_ in a canalized-reaction consuming glutathione, producing H_2_O. This system correctly balances ROS production under physiological conditions. However, the imbalance between ROS and antioxidant defense in favour of ROS overproduction generates oxidative stress. A metabolic state able to produce oxidative stress in different organs and typically observed in developed societies is represented by overfeeding, especially in terms of a high consumption of saturated lipids. Several research groups, including our group, have shown that HFD induces alterations in the serum lipid profile, as well as increasing both the body weight and lipid depots in adipose tissue and ectopic sites [17,29,60,61]. It is noteworthy that a high dietary fat intake has been shown to be associated with oxidative stress induction in different experimental models and tissues [29,31,32,62,63], suggesting that fatty acids represent a critical point in the development of metabolic disorders associated with obesity. Moreover, as stated above, another environmental condition that may induce oxidative stress in different tissues and organs is the exposure to environmental pollutants [15,16]. Our experimental results have shown that DDE induces oxidative damage, cellular death, and tissular injury [33,34]. In the following sections, we focus on the HFD and/or environmental pollutant, mainly DDE, effect on mitochondrial dysfunction and oxidative stress in the liver and testis.

## 3. Physiological Adaption to HFD and DDE in the Liver

### 3.1. HFD Induces Hepatic Fat Accumulation and Cellular Stress Targeting Mitochondria

It is well-known that HFD induces obesity and alters the serum lipid profile [64]. The excess fatty depots positively correlate with adipose tissue inflammation, insulin resistance and metabolic disorders, such as hepatic steatosis [65,66]. Fat depots in the adipose tissue remain unchanged over time under conditions of adequate nutritional intake. On the other hand, an imbalance in macronutrient percentage towards lipid intake elicits an increase in lipid depots and metabolic changes in energetic substrates utilization in both adipose tissue and ectopic fat depots, such as the liver. In fact, literature data reported that HFD increases the cellular capacity to utilize fatty acids by activating a pool of genes directly involved in the storage, mobilization, transport, and oxidation of fats [67,68,69,70]. In accordance, several studies have suggested that HFD-fed animals adapt their cellular metabolism, modulating the expression of nuclear genes mainly involved in lipid oxidation, such as the peroxisome proliferator-activated receptor (PPAR). PPAR family members (α, δ, γ) act as metabolic regulators in cells. PPARα has been shown to be involved in liver fatty acid metabolism [71]. It was found that PPARα regulates hepatic fatty acid transporters and carnitine palmitoyltransferase 1 and 2 (CPT-1 and -2) activity, increasing fatty acid utilization by mitochondria [72,73]. In fact, PPARα targets genes coding β-oxidative enzymes to regulate fatty acid utilization, [72]. This mechanism represents an important point of metabolic adaptation particularly used in a fasting condition, where fatty acid utilization generates ketone bodies used by tissues to produce energy [74]. Several researchers have also evidenced a role of PPARα in non-alcoholic fatty liver disease (NAFLD) models [29,75,76,77]. Lipid deposition and body mass increased in HFD-fed animals, resulting in increased plasma fatty free acids levels and hepatic PPARα expression [75]. Indeed, it has been shown that HFD increased hepatic PPARα levels and its nuclear localization [29]. It has also been reported that PPARs are able to induce mitochondrial uncoupling by stimulating mitochondrial UCPs in different organs [29,76,77], with a resulting increase in mitochondrial energy expenditure not coupled with ATP formation.

Under the condition of change in lipid metabolism towards fatty acid oxidation, ketone bodies (β-hydroxybutyrate and acetoacetate) are significantly increased in the serum [78], suggesting that ketogenesis represents a metabolic adaptation under conditions of high-fat feeding, as well as in insulin resistance and fasting [79]. Moreover, it was recently reported that ketogenesis could represent an important adaptive mechanism to prevent NAFLD in an animal model [74]. In fact, in mice with induced-insufficient ketogenesis, HFD produced severe hepatic injury, inflammation, impaired hepatic gluconeogenesis, and deranged hepatic tricarboxylic acid (TCA) cycle intermediate concentrations, suggesting that ketone bodies function as hepatic metabolic regulators, playing a possible central role in fatty liver disease prevention [80].

**Figure 1:**
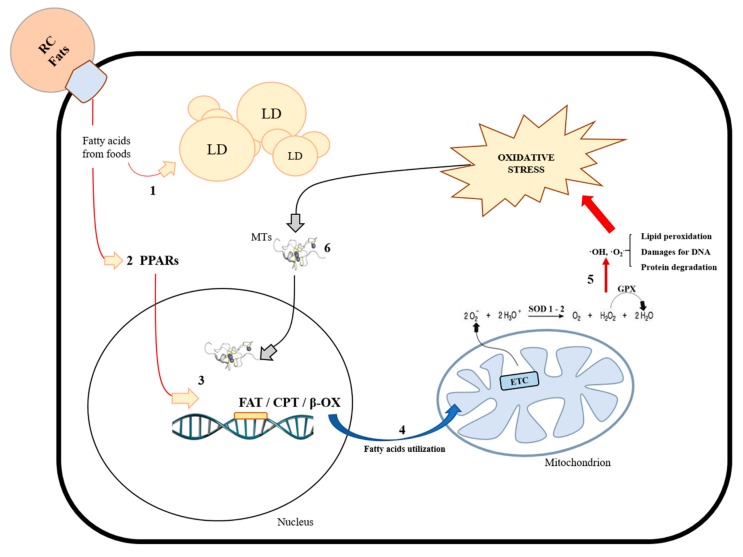
Effect of a high-fat diet (HFD) in hepatocytes. Dietary fats reach the liver from the blood vessels by remnant chylomicrons (RC) and enter the hepatocytes. In cell cytosol, elongated fatty acids can be in part accumulated in the form of lipid droplets (1) or used as energetic substrates. PPAR family members (2) play a key role in lipid catabolism activation by inducing the expression of genes (3) involved in the transport and use of fatty acids by mitochondria (4). As a consequence, increased mitochondrial activity produces elevated reactive oxygen species (ROS) levels that, if not adequately counterbalanced by endogenous antioxidant defenses, induce oxidative damage on lipids, proteins, and DNA (5), with negative consequences on the mitochondrial function. Under these metabolic conditions, nuclear transcriptional activity increases to adapt cellular metabolism. Then, MTs localize in the nucleus (6) to donate or chelate metals or to protect DNA from oxidative damage. RC: remnant chylomicrons; Fats: fatty acids; LD: lipid droplets; PPARs: peroxisome proliferator-activated receptors; FAT: fatty acid transporters; CPT: carnitine palmitoyl-transferase; β-OX: beta oxidation; ETC: electron transport chain; H_2_O_2_: hydrogen peroxide; OH^•^, O_2_^•−^: reactive oxygen species; SOD1-2: superoxide dismutase; GPX: glutathione peroxidase; MTs: metallothioneins.

Several studies have suggested that alterations in mitochondrial function and oxidative stress play a key role in the onset of diet-induced hepatic steatosis [26,81,82,83,84]. In our recent works, we analysed mitochondrial fatty acid oxidation, oxidative stress, and antioxidant defenses in HFD-fed rats [34,85]. The results showed that HFD increased the beta-oxidation (β-ox) rate and carnitine palmitoyl-CoA transferase (CPT) activity, confirming that the liver increased the utilization of lipids as energetic substrates during overfeeding [34]. In addition, the excess fats introduced with food elicited lipid accumulation in the hepatocytes and increased oxidative damage in terms of the accumulation of both malondialdehyde (MDA) and oxidized glutathione (GSSG) in the tissue [34]. This cellular stress is probably due to the excess ROS produced at a mitochondrial level by increased hepatic lipid catabolism. It is noteworthy that the analyses of the antioxidant system suggested that the liver adapts its metabolism, at least in part, by stimulating both SODs and GPx protein levels and activities [34]. Therefore, the stimulation of antioxidant systems may play a key role in the control of the oxidative stress level. In addition, our study of the antioxidant system was extended to other classes of proteins with non-specific antioxidant activity, such as the small intracellular proteins known as metallothioneins (MTs) [85]. Experimental studies have shown that MTs quench hydroxyl radicals in vitro [86]. Unexpectedly, we found a reduction in MTs gene expression and protein synthesis in diet-induced hepatic steatosis and oxidative stress [85]. However, histochemical experiments highlighted increases in the nuclear localization of MTs, which was also confirmed by western blot analysis of a liver nuclear fraction [85]. These data, according to the literature, suggested a possible role of MTs at a nuclear level in oxidative stress conditions. MTs may play a key role in protecting DNA from hydroxyl radicals, in donating metals for several enzymes, or in chelating zinc from transcription factors such as zinc finger proteins [85,87,88,89,90,91]. In order to limit cellular stress, hepatocytes use an additional strategy to directly control ROS generation at a mitochondrial level through the uncoupling of mitochondrial respiration from the oxidative phosphorylation process. This mechanism is controlled by a family of protein carriers known as uncoupling proteins (UCPs), which are composed of different members that are expressed in different tissues: UCP1 (mainly in brown adipose tissue), UCP3 (mainly in muscle), and UCP4-5 (mainly in the brain) [92,93,94]. We analysed the member of the UCPs family that was found to be ubiquitously expressed: UCP2 [95]. This protein is essentially involved in the control of ROS production [96,97], representing the isoform that mainly differs in function compared to the other UCP isoforms. In our experimental conditions, HFD induced UCP2 gene expression and protein synthesis, suggesting its possible involvement in fatty liver metabolism [34]. Nevertheless, all the activated mechanisms seemed to be unable to completely counteract hepatic oxidative damage induced by fats. The liver seemed to fail to properly balance the excess ROS due to the large amount of lipids. Therefore, the liver accumulated fat and proceeded towards oxidative damage and steatosis, as also shown by the increase in serum levels of transaminase, markers of hepatic injury [34]. We have summarized the observed effects induced by HFD in hepatocytes in Figure 1.

### 3.2. DDE Exposure: Hepatic Mechanisms Used to Counteract Liver Damage

In recent years, experimental evidence showed that environmental contaminants are also involved in inducing TASH, eliciting fatty liver injury in non-obese people exposed to chemicals and/or xenobiotics [98,99]. In fact, several environmental pollutants induce metabolic disorders in the cells [100]. Phthalates, organochlorines, and other environmental chemicals have been shown to be involved in ROS accumulation and apoptosis onset [15,101]. Under a stress condition, cells activate compensatory mechanisms that include the modulation of gene expression. In particular, phthalates can induce hepatic toxicity through PPARs transactivation, causing uncontrolled proliferation and hepatic tumorigenesis development [102]. In addition, PPARs have been shown to be directly involved in the regulation of cellular toxicity induced by air, water, and food pollutants [103]. Air pollutant exposure reduced PPARs levels, producing tissular injury in terms of hepatic inflammation, endoplasmic reticulum stress [104], and steatosis. In fact, PPARs not only regulate cellular proliferation, but also play an anti-inflammatory role and maintain lipid homeostasis in Kupffer cells, hepatocytes, and stellate cells [105]. Moreover, the induction of PPARγ gene expression in the presence of DDT has been seen in human mesenchymal stem cells, [106] suggesting that PPARs, as well as other nuclear receptors, are involved in the cellular response to toxicant.

In addition, chronic exposure to environmental toxic substances, including DDT and DDE, has been shown to induce DNA damage in different models in vivo and in vitro [107,108,109]. However, cells adapt their metabolic state under a stress condition by activating a series of signals that include the expression of genes involved in metabolic homeostasis, such as the up-regulation of antioxidant systems [110] and the increase in mitochondrial activity to produce the additional ATP required by the cellular function [111,112]. However, the increased mitochondrial activity is associated with increased ROS levels that, if not correctly balanced, generate oxidative stress that in turn elicits damage to macromolecules, genomic instability, and mitochondrial/cellular dysfunction [40].

Recent scientific findings showed that DDE induces oxidative stress, mitochondrial impairment, and cellular oxidative damage [15,16,34,35]. In our study, chronic exposure to a non-toxic DDE dose, associated with a standard diet regimen (N + DDE group), did not produce changes in terms of the serum lipid profile and fatty acid deposition in the tissues compared to the control group [34]. However, hepatic morphological alterations were histologically detected in terms of eosinophilic cells around the principal vessels, perivascular cellular vacuolization, and inflammation [34,85]. These morphological changes were probably due to oxidative stress induced by exposure to pesticides, which produce cellular modification typically associated with the onset of TASH [98]. Experimental data showed increased hepatic levels of MDA and GSSG, confirming the role of DDE in oxidative stress generation [34]. However, the mechanisms by which DDE induces ROS incrementation are not yet clear, but the main effects of DDE on hepatic cells involve the cytochrome P450 (CYP450) activity, used in the detoxification path [113]. It has been shown that DDE activates CYP450 gene family transcription, mainly CYP450 2B and 3A, by interacting with the constitutive androstane receptor (CAR) and pregnane X receptor (PXR) [114]. It has also been shown that pesticide can directly affect the mitochondrial electron transport chain inducing functional impairment [115,116]. In our experimental model, we found increases in both mitochondrial β-ox and CPT system activity [34]. These findings suggest that mitochondria play a pivotal role in the adaptation to DDE exposure by increasing fat catabolism to face the increased cellular energy needs for the hepatic detoxification processes. Together with hepatic oxidative damage, the principal antioxidant enzymes, namely SODs and GPx, were found to be stimulated in terms of protein synthesis and enzymatic activity [34]. In particular, also in this context, mitochondria played a central metabolic role in controlling the cellular oxidative balance. In fact, a strong stimulation of the mitochondrial SOD isoform (Mn-SOD, well-known as SOD2), was found that represents one of the most important mitochondrial defenses against ROS [34]. In addition, a reduction in MT levels was found in DDE-treated rats, as was found in a similar way in HFD-treated rats [85].

**Figure 2:**
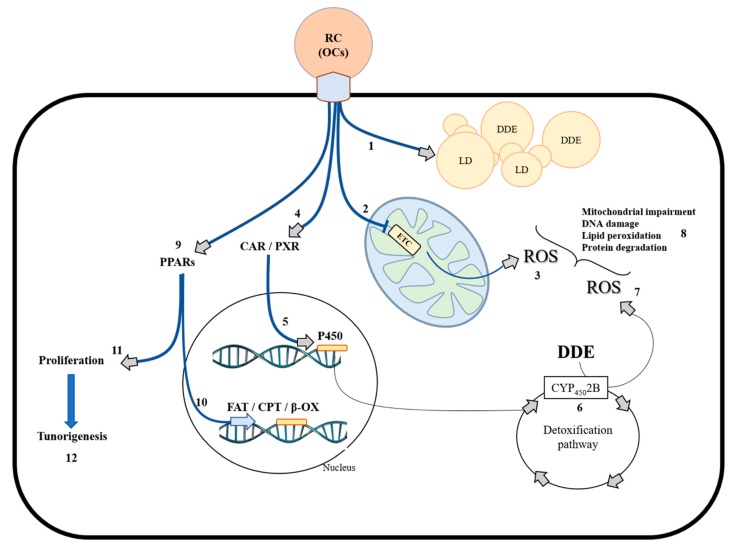
Principal mechanisms stimulated by organochlorinated compounds. Organochlorinated compounds (OCs), including DDE accumulated in contaminated foods, reach the liver from the blood vessels by remnant chylomicrons. In the cells, OCs can be, in part, stored in the lipid droplets (1). In addition, OCs can act on mitochondria, partially inhibiting the electron transport chain (2) and inducing mitochondrial ROS production (3). Moreover, OCs such as DDE can interact with CAR and PXR receptors (4), inducing expression of the P450 gene family (5) for detoxification (6). However, the detoxification path augments ROS levels (7). These mechanisms, if not effectively controlled by antioxidant defenses, produce oxidative damage in macromolecules (8). In addition, OCs can modulate transcriptional activity through PPAR family members (9), modulating fatty acid metabolism (10) and cellular proliferation (11). Uncontrolled proliferative mechanisms induced by OCs can lead to tumorigenesis (12). RC: remnant chylomicrons; OCs: organochlorinated compounds; DDE: Dichlorodiphenyldichloroethylene; LD: lipid droplets; ETC: electron transport chain; CAR: constitutive androstane receptor; PXR: pregnane X receptor; PPARs: peroxisome proliferator-activated receptors; FAT: fatty acid transporters; CPT: carnitine palmitoyl-transferase; β-OX: beta oxidation; P450: cytochrome P450; ROS: reactive oxygen species.

Moreover, we also demonstrated strong MTs nuclear localization in the DDE-treated liver, confirming a physiological nuclear role of MTs in an oxidative stress condition [85]. The mitochondrial role in the hepatic adaptation to pesticide-induced oxidative stress has become increasingly evident during research. In fact, alongside the high SOD2 level, hepatocytes from DDE-treated rats showed high UCP2 gene expression and protein synthesis [34]. Probably, both SOD2 and UCP2 play a cooperative role in response to DDE at a mitochondrial level. The electron transport chain produces a superoxide anion, which is rapidly converted into H_2_O_2_ by SOD2. However, to avoid an excessive accumulation of H_2_O_2_ at a mitochondrial and cellular level, mitochondrial uncoupling is induced in order to reduce superoxide production by mitochondrial activity. This effect, associated with the increase in GPx activity observed in the liver, probably contributes to not exacerbating the oxidative damage and hepatic injury, that, in terms of the serum transaminases level, is comparable to that induced by HFD treatment [34]. The hepatocyte response to OCs, namely DDE, has been summarized in Figure 2.

### 3.3. Dietary Fats and DDE Showed No Synergic Effect in Hepatic Stress Generation and Damage

The simultaneous exposure to HFD and the environmental pollutant DDE (D + DDE group), represents a very interesting topic to be addressed and we showed that pesticide affects hepatic metabolism differently, depending on the different dietary treatment (low-fat or high-fat diet) [34]. At a systemic level, D + DDE animals presented alterations in the serum lipid profile in terms of triglycerides and cholesterol levels, but serum triglyceride levels were found to be lower compared to the levels in D animals. In line with this result, hepatic lipid depots were found to be lower in D + DDE than in the D group. Moreover, mitochondrial β-ox and CPT system activities were increased to the same extent as in N + DDE-treated animals [34]. It can be suggested that the lower levels in serum triglycerides and hepatic lipid depots could be at least in part due to the increased hepatic fatty acid utilization required to produce energy to cope with the increase in the detoxification pathway. In fact, CYP450 2B protein content was found to be increased in the D + DDE and N + DDE groups [34]. Hepatic morphological changes showed several inflammatory foci, eosinophilic cells, and hepatocytes vacuolization around blood vessels [34]. Oxidative stress occurred in D + DDE animals with the accumulation of MDA and GSSG in the liver, as was found in the N + DDE animal group. We did not observe any additional oxidative damage in the D + DDE group compared to both the N + DDE and D group, as it can be expected by potential additional pro-oxidant effects due to the simultaneous exposure to two different pro-oxidant stimuli, namely HFD and DDE [34]. Therefore, it can be suggested that no synergic effects on hepatic stress and damage occurred, when pesticide exposure was simultaneous to high-fat diet treatment. Considering the hydrophobic nature of this pesticide, in the condition of lipid accumulation in adipose tissue and in ectopic sites observed in D and D + DDE groups, it can be hypothesized that a part of DDE remains stored in lipid depots in an inert form. Therefore, the adaptive metabolic responses in the D + DDE group were mainly induced by dietary fats and only in part by the pesticide, and no synergic effect of dietary fat and pesticide on oxidative stress was observed in D + DDE. Increased SOD and GPx activities and reduced MT levels were also found in the D + DDE group [34,85]. However, in this case, MTs maintained nuclear localization in the hepatocytes as in D group [85], whereas SOD2 activity was found to be intermediate between D and N + DDE [34], suggesting that dietary fats limit, at least in part, DDE activity. Moreover, mitochondrial levels of UCP2 in terms of gene expression and protein synthesis in the D + DDE group were intermediate between D and N + DDE groups. Finally, serum transaminase levels were found to be increased, as in D and N + DDE groups, indicating no further liver damage in the D + DDE group as a consequence of the sum of the responses to the two different stimuli [34].

## 4. Physiological Adaption to HFD and DDE in the Testis

### 4.1. HFD Alters Testicular Function and Affects Hormonal Homeostasis

**Figure 3:**
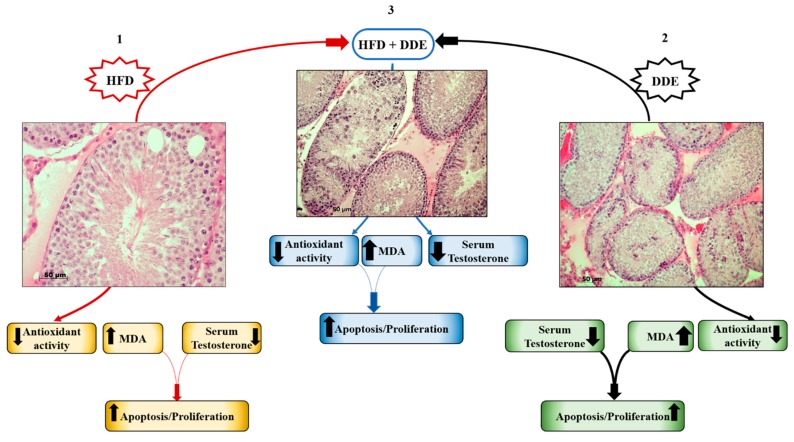
Effects of singular and simultaneous exposure to a high-fat diet (HFD) and/or dichlorodiphenyldichloroethylene (DDE) in testis. HFD (1) reduced antioxidant activity and generated oxidative stress. Increases in the apoptotic rate were found. In addition, dietary fats reduced serum testosterone levels, altering the hormonal balance. Both DDE exposure (2) and simultaneous HFD and DDE exposure (3) affected antioxidant system activity, which was strongly reduced. Oxidative damage further increased, and to a much greater extent in DDE-treated animals (2). Moreover, a trigger of apoptosis, tubular damage, and a reduction of testosterone levels were detected. To counteract cellular death, proliferation was stimulated in a similar way in HFD (1) and HFD + DDE (3), and was further increased in DDE (2), suggesting the different effects of pesticides at a cellular level.

Different analyses were conducted to evaluate the effects of dietary fats on testicular function and morphology [33]. The results showed that HFD alters the antioxidant system and induces oxidative damage. In fact, GPx activity reduction and lipid peroxidation induction with MDA accumulation were observed in the tissue [33]. Moreover, as observed in the liver, a reduction in MTs gene expression and protein synthesis occurred [85]. As a consequence of oxidative injury, morphological alterations were detected in the seminiferous epithelium [33]. Large spaces among germinal cells as well as cytoplasmic vacuolization in germinal and Sertoli cells were observed [33], probably due to lipid and/or fluid accumulation [117]. Activation of apoptotic stimuli was observed under the condition of oxidative stress [33]. Normally, testicular germ cell apoptosis represents a physiological process playing a key role in removing abnormal spermatozoa during spermatogenesis [118,119]. On the other hand, in a non-physiological metabolic state, such as an oxidative stress condition, the high apoptotic rate could be associated with male subfertility. In obese subjects, the onset of male subfertility with changes in sperm parameters, motility and counting, can occur due to the high-fat induced cellular oxidative stress [120,121,122]. In our study, we showed an increase in Bcl2 associated with X protein (BAX) levels and activated caspase 3 (casp-3) cell immunoreactivity in HFD-fed animals, where an apoptosis trigger was observed [33]. Moreover, HFD was also found to be involved in hormonal disorder generation, which probably represents a key point in testis functional alteration [123]. In fact, HFD-fed animals showed a reduction in testicular androgen receptor (AR) and serum testosterone (T) levels [33]. Noteworthy, we also found a stimulation of cellular proliferation through the increase in the content of proliferating cell nuclear antigen (PCNA). This increase may be involved in cellular mechanisms useful to counteract oxidative stress-induced cellular death in the testis. We have summarized the effect of HFD in the testis in Figure 3.

### 4.2. DDE Negatively Affects Spermatogenesis and Testicular Function

It is well-known the effect of PCBs on the male reproductive system [124,125]. DDE acts as antiandrogenic chemical by interacting with androgen receptors [12]. In our work, we analysed the effect of DDE on the testicular function to evaluate the in vivo adaptive mechanisms during chronical exposure to a non-toxic dose [33]. We confirmed the pro-oxidant role of the pesticides with a reduction in both total SODs and GPx activities, as well as in MTs gene expression and protein synthesis [33], in accordance with previous finding [16]. In our study, antioxidant impairment in DDE-treated rats induced the highest MDA levels compared to the other experimental groups [33]. As a consequence, morphological alterations were detected at different levels. A strong disorganization of the seminiferous epithelium was observed in some tubules, where it was no longer possible to detect the typical cell–cell associations. Moreover, eosinophilic cells with pyknotic nuclei were detected. The lumen of severely altered tubules was rich in non-differentiated cells [33]. This observation suggested that cell-cell adhesion was lost, and non-differentiated cells were released in the lumen without completing spermatogenesis. A similar finding was also reported in further in vivo animal models with exposure to other toxic chemical species [126,127,128]. In DDE-treated rats, the highest BAX protein levels and casp-3 cell immunoreactivity were detected compared to N and D animals, associated with the induction of hormonal disorders in terms of reduced testicle AR protein levels and serum T [33]. Noteworthy, a stimulation of cell proliferation was observed in association with increases in the apoptotic rate, as also observed in HFD-fed group [33]. The highest PCNA levels observed in DDE-treated group [33] was in line with previous findings showing that DDE is able to induce cellular proliferation through oxidative stress [129] (Figure 3).

### 4.3. Simultaneous Exposure to HFD + DDE did not Show a Synergic Effect on Testicular Function

In the experimental group exposed to both HFD and DDE, the effects of DDE in the testis did not show synergistic effects with HFD [33], as described for the liver above [34]. The results obtained in D + DDE group showed a very similar effect to that of DDE-treated animals in terms of morphological alterations, antioxidant impairment, apoptosis, and hormonal changes [33]. On the other hand, MDA levels were found to be intermediate between D and N + DDE groups. According to the lower oxidative damage vs. N + DDE, we showed a reduced MTs gene expression and protein synthesis in D + DDE animals, as in the D group, suggesting the transcriptional regulation of MTs, depending on the tissular oxidative stress level. Moreover, cellular-induced proliferation didn’t seem to be stimulated as observed in N + DDE, but the response was more similar to that observed in the D group [33]. These findings suggested that DDE has less impact when administrated together with HFD, in accordance with the lipid dissolving theory which hypothesizes that DDE storage in body fat deposits induces a decrease in DDE effects In Figure 3, we have schematized the responses to HFD and/or DDE in the testis.

## 5. Conclusions

In conclusion, our research confirmed the literature on HFD and DDE effects in terms of oxidative stress associated with changes in lipid metabolism, mainly in the liver.

Hepatic adaptations to dietary fats and pesticides induced a general control of hepatic injury in which mitochondrial function and cellular metabolism were directly involved. Antioxidant system stimulation and mitochondrial UCP2 up-regulation seemed to play a potential protective role in counteracting ROS overproduction, mainly in the presence of environmental pollutants.

In the testis, HFD altered the oxidative balance and induced lipotoxicity, cellular death and apoptosis. On the other hand, DDE generated the highest tissular alterations and dysfunctions with impairment of spermatogenesis under the conditions of both individual or simultaneous exposure to pesticide and dietary fats, with a worse redox state when pesticide exposure was not associated with a high fat consumption.

With the limitation that further studies are needed to better identify the mitochondrial role in response to environmental stimuli, such as pollutants and chronic high-fat feeding, our experimental research summarized in the present review was the first to address the topic of mitochondrial and oxidative stress responses to chronic simultaneous exposure to both environmental stimuli in different organs. Given that, in modern obesogenic society, we are frequently exposed to both a high-fat diet and several environmental pollutants with consequently increased risks of related metabolic disease, studies on mitochondrial involvement in the cellular response to these environmental stimuli can be useful in understanding the adaptive mitochondrial response required to maintain a healthy condition and, therefore, to target mitochondria in the prevention and therapy of environmental stimuli-induced metabolic diseases.
